# Study of the response of a single-dof dynamic system under stationary non-Gaussian random loads aimed at fatigue life assessment

**DOI:** 10.1016/j.heliyon.2024.e30832

**Published:** 2024-05-14

**Authors:** Michele Sgamma, Massimiliano Palmieri, Michele Barsanti, Francesco Bucchi, Filippo Cianetti, Francesco Frendo

**Affiliations:** a Department of Civil and Industrial Engineering, University of Pisa, Pisa, Italy; b Industrial Engineering Department, University of Perugia, Perugia, Italy

**Keywords:** Fatigue analysis, Random loads, Non-Gaussian signals, Frequency domain, Computational cost

## Abstract

Fatigue assessment of components subjected to random loads is a challenging task both due to the variability in amplitude and frequency of the loads and for the computational times required to perform classical time domain fatigue analysis. The frequency domain approach to fatigue life assessment offers a solution by utilizing the power spectral density of the random load, requiring minimal computational effort. However, frequency domain methods are limited to stationary Gaussian signals, while real-world loads often exhibit non-Gaussian characteristics. Previous research proposed formulas to extend frequency domain methods to non-Gaussian cases, but they require knowledge of the parameters related to non-Gaussianity of the component's stress (skewness and kurtosis), which would require a time domain analysis of the stress history on the component and a strong reduction of the computational advantages. This paper aims to address this gap by conducting an extensive campaign of numerical simulations to evaluate the influence of various parameters on the degree of non-Gaussianity of the response of a system. A single-dof mass-spring-damper system was subjected to non-Gaussian random loads of different natures, and the response is analyzed to determine the values of skewness and kurtosis. The study investigated the influence on non-normality indexes of the system's output of several input parameters, which include both the characteristics of the input load and the properties of the dynamic system. The findings contribute to a better understanding of non-Gaussianity in dynamic systems and pave the way for conducting efficient fatigue analyses in the frequency domain. Future work will extend the study to non-stationary random loads, further advancing the understanding of non-Gaussianity and non-stationarity in dynamic systems.

## Highlights

• The damping coefficient significantly impacts the output skewness and kurtosis of the system response to non-Gaussian loads.
• Systems with very low damping give Gaussian outputs regardless of the input's non-Gaussianity indices.
• The ratio between the bandwidth of the load and the system's eigenfrequency acts as an invariant of the problem.
• The findings have implications for time-saving frequency fatigue analyses in structural engineering applications.

## Graphical abstract

**Figure:**
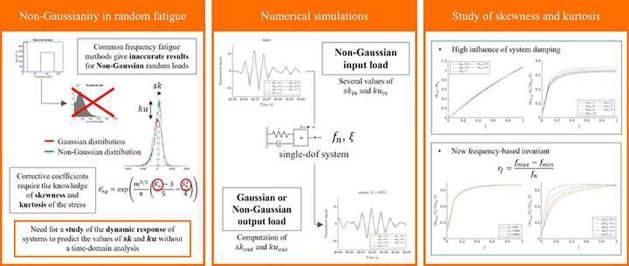

## 1 Introduction

Fatigue is a common occurrence in materials and machine components that are subjected to repeated loads, which can cause damage and weaken the parts over time. In the case of random loading, which lacks a predictable pattern, it is especially difficult to estimate the fatigue life of components. Actually, stochastic stresses often occur during components' service life, such as those experienced by mechanical and electronic components in vehicles and aircraft, thus making fatigue life assessment due to random loads an important topic for many engineers and designers. The effects of random loads on materials are complex and hard to predict due to their variability both in amplitude and frequency. Experimental measurements can determine the load profiles of stochastic loads, but simulation using Finite Element (FE) and Multibody (MB) models is often impractical due to prohibitively long computation times. The frequency domain approach to fatigue life assessment offers a solution to this problem by using the Power Spectral Density (PSD) of the random load, which requires minimal computational effort. This approach evaluates the Probability Density Function (PDF) of cycle amplitudes directly from the PSD of the stress, translating the results of a counting method from the time domain to the frequency domain. The rainflow method [1] is considered the most reliable time-domain cycle counting method, and several other methods have been developed in the literature to approximate its results for the marginal probability density function of the amplitudes of the cycles and for resulting overall damage under certain simplifying assumptions, that are the use of stationary, ergodic, and Gaussian signals. Bendat [2] proposed a Rayleigh function to estimate the amplitude probability density function of rainflow cycles for narrow-band signals. However, the accuracy of this distribution reduces significantly as the bandwidth of the random load increases. To apply Bendat's theory to wide-band signals, various authors suggested different corrective coefficients to adjust Bendat's damage estimation and obtain a corrected total damage value [3], [4], [5], [6]. Other authors proposed new formulations of the marginal amplitude PDF of rainflow count by combining different basic distribution functions (e.g., exponential, Rayleigh, Weibull) [7], [8], [9]. Dirlik [7] obtained the most significant results by conducting a large number of numerical simulations and proposing a formula based on the combination of an exponential distribution with two Rayleigh distributions. This formula achieved a low error approximation of the marginal amplitude pdf of rainflow count for a wide range of signal bandwidth.

Although these frequency methods are a good approximation, they still have significant limitations that can constrain their applicability in real-world scenarios [10], [11], [12], [13]. One major limitation is their reliance on restrictive assumptions, including the assumptions of ergodicity, steady-state conditions, and Gaussian signals. These assumptions are particularly hindering as non-Gaussian random loads are common in many engineering applications, including wind turbines, offshore structures, and vehicular, railway, and aeronautical applications [14], [15], [16], [17]. Gaussian signals are completely described by their mean value and standard deviation, which are the first two moments of their distribution, while non-Gaussian signals require the definition of two additional higher-order moments: skewness and kurtosis. Skewness is a measure of the asymmetry of the distribution with respect to its mean value, while kurtosis is related to the concentration of the distribution around the mean value. A Gaussian signal has a skewness value of zero and a kurtosis value of 3.

To expand the applicability of methods designed for Gaussian loads to non-Gaussian loads, several corrective formulas have been proposed in the literature. These formulas estimate the damage caused by non-Gaussian loads $D_{\mathrm{ng}}$ by multiplying the expected damage $D_{g}$ from a Gaussian load with the same power spectral density as the actual load by a corrective coefficient $\lambda_{\mathrm{ng}}$ [18], [19], [20], [21], [22], [23], [24]. However, the computation of such coefficients requires knowledge of the skewness and kurtosis of the system's response in terms of stresses, which requires analyzing the stress history in the time domain. This negates the computational benefits of performing an entirely frequency-based analysis [25]. Therefore, further research is needed to study mechanical system responses, in terms of stress, to non-Gaussian inputs, enabling prediction of the response's skewness and kurtosis directly from the probability distribution function of the input signal and system parameters.

Rizzi et al. [26] and Kihm et al. [27] studied the effects of kurtosis and skewness on the fatigue life of linear and non-linear systems, and they demonstrated that non-Gaussian loads in linear systems result in Gaussian responses because the Central Limit Theorem (CLT) is observed when the period of the system impulse response is significantly longer than the rate of peak loading. On the other hand, the CLT was broken, and the response might not be Gaussian, if the timescale of the loading's peaks is equal to or longer than the period of the system's impulse response. Palmieri et al. [28] carried out an experimental campaign to investigate the dynamic response of a system to both stationary and non-stationary non-Gaussian signals. The results suggest that the non-Gaussianity of the system's response does not arise when the input load is non-Gaussian and stationary, as the system response remains Gaussian. Conversely, when the input load is non-Gaussian and non-stationary, the system's output maintains a level of non-Gaussianity similar to that of the input load. The same authors performed additional numerical analysis on a single-dof system to generalize the results obtained from the experimental campaign [29]. The tests, performed for various values of kurtosis of the input signal, confirmed what was observed for non-stationary and non-Gaussian signals. Conversely, for stationary non-Gaussian loads, results showed a significant dependence of the system's response characteristics on the damping value of the system. In fact, the response is Gaussian only when the damping is very low, while for higher damping values the system's response remains non-Gaussian. More recently, some authors proposed to adopt high-order spectrum to quantify the kurtosis transmission rate [30], [31]. Therefore, it is clear that there is potential for further study of the response of a mechanical system to inputs characterized by different degrees of non-Gaussianity, so that the skewness and kurtosis values of the stresses on a component can be predicted directly from the properties of the probability distribution of the input load.

In this regard, in this work, an extensive campaign of numerical simulations was conducted to evaluate the influence of various parameters on the degree of non-Gaussianity of the response, leaving the non-stationary case for further investigation. Specifically, a single-dof mass-spring-damper system was subjected to non-Gaussian random loads of different natures, and the response is analyzed to determine the values of the two non-normality indexes of interest: skewness and kurtosis. The variation of the kurtosis and skewness of the output was evaluated by considering the effect of several input parameters, which include both the input load (its frequency content and the properties of its probability distribution) and the properties of the dynamic system (eigenfrequency and damping).

## 2 Fatigue in the frequency domain

### 2.1 Gaussian random loads

The input for the frequency approach to fatigue life estimation is represented by the stress Power Spectral Density (PSD). The PSD is a scalar function that describes how the power of the signal is distributed across various frequencies and, especially in the case of stationary, ergodic, and Gaussian signals, it provides the most concise and complete representation of a stochastic process. Some information about the properties of a signal in the time domain can be determined based on the spectral moments of the PSD. The spectral moment of order *n* can be obtained from Equation (1), where *ω* is expressed in rad s^−1^

(1) $$\begin{array}{ccc} \lambda_{n}\; & =\; & \int_{0}^{\infty }\omega^{n}\;PSD(\omega )\;d\omega \end{array}$$

The zero-order spectral moment is equal to the variance of a correspondent time-signal. Two more important time-domain properties, namely the mean up-crossing rate $f_{0}$ and the peak occurrence $f_{p}$ can be related to the PSD through Rice's formulas [32] (equation (2)). The ratio between these parameters defines the Irregularity Factor *γ* (equation (3)), which is one of the most important parameters for frequency fatigue analysis since it gives information on whether the signal is narrow-band (γ→1) or wide-band (γ→0)

(2) $$\begin{array}{cccccc} f_{0}\; & =\; & \frac{1}{2\pi }\sqrt{\frac{\lambda_{2}}{\lambda_{0}}}\; & \;f_{p}\; & =\; & \frac{1}{2\pi }\sqrt{\frac{\lambda_{4}}{\lambda_{2}}} \end{array}$$

(3) $$\begin{array}{ccccc} \gamma \; & =\; & \frac{f_{0}}{f_{p}}\; & =\; & \frac{\lambda_{2}}{\sqrt{\lambda_{0}\;\lambda_{4}}} \end{array}$$

Narrow-band signals have a single dominant frequency, and thus they have a peak rate very close to the mean up-crossing rate. This represents an interesting case, since for such signals Bendat's theory [2] provides the most important theoretical result for frequency translation of time-domain cycle counting methods. From the assumptions of zero mean value of the whole signal and narrow-band signal, Bendat deduced that the mean value of the cycles can be considered irrelevant and consequently the amplitude of each cycle can be considered equal to the peak of the cycle signal. His study, therefore, focuses on estimating only the marginal PDF of cycle amplitudes, which turns out to be equal to the pdf of the signal peaks and is described by a Rayleigh distribution only dependent on the zero-order moment of the PSD.

(4) $$\begin{array}{ccc} p(S)\; & =\; & \frac{S}{\lambda_{0}}\;e^{-\frac{S^{2}}{2\lambda_{0}}} \end{array}$$

The number of peaks in a wide-band signal is higher than the mean number of up-crossings; this causes a decrease in the irregularity factor values as the frequency span of the signal PSD increases. In this way, the assumptions behind Bendat's theory are broken, and in the case of wide-band signals, it overestimates the total fatigue damage and leads to results that are no longer acceptable [33], [34]. Several authors [3], [4], [5], [6], [7], [8], [9] tried in various ways to overcome this problem and to extend the theory to wideband signals, but the best results were obtained by Dirlik [7], who developed, through a large number of numerical simulations, the formula in equation (5), which represents the best frequency approximation of the results obtained in the time domain.

(5) $$\begin{array}{ccc} p(z)\; & =\; & \frac{\frac{D_{1}}{Q}\;e^{-z/Q}+\frac{D_{2}\;z}{R^{2}}\;e^{-z^{2}/(2R^{2})}+D_{3}\;z\;e^{-z^{2}/2}}{2\sqrt{\lambda_{0}}}\\ \text{Wherẹ:}\; & \; & \\ D_{1}\; & =\; & \frac{2(x_{m}-\gamma^{2})}{1+\gamma^{2}}\\ D_{2}\; & =\; & \frac{1-\gamma -D_{1}+D_{1}^{2}}{1-R}\\ D_{3}\; & =\; & 1-D_{1}-D_{2}\\ R\; & =\; & \frac{\gamma -x_{m}-D_{1}^{2}}{1-\gamma -D_{1}+D_{1}^{2}}\\ Q\; & =\; & 1.25\;\frac{\gamma -D_{3}-D_{2}R}{D_{1}}\\ x_{m}\; & =\; & \frac{\lambda_{1}}{\lambda_{0}}\sqrt{\frac{\lambda_{2}}{\lambda_{4}}} \end{array}$$

### 2.2 Non-Gaussian random loads

A non-Gaussian random load refers to a type of load that deviates from a Gaussian or normal distribution, which is commonly characterized by a symmetrical bell-shaped curve that represents the probability of occurrence of a certain value. Non-Gaussian random loads are typically described as having heavier tails, meaning that there is a greater probability of extreme values occurring in comparison to a Gaussian distribution. This complexity makes non-Gaussian random loads more difficult to analyze and predict, as extreme values can have a substantial impact on structures and components. In contrast to a Gaussian load, whose probability distribution can be defined by only the first two central moments (mean value and standard deviation of the signal), non-Gaussian loads would require the definition of an infinite number of central moments to characterize the probability distribution function. However, previous studies have suggested that the third- and fourth-order moments, which are normalized with respect to the standard deviation and referred to as skewness and kurtosis, respectively, have the most significant effect on the distribution tails (and thus the impact of the load on the fatigue damage of the components) [35]. In Gaussian loading, the skewness value is zero, and the kurtosis value is equal to 3.

For these reasons, the frequency methods described in the previous section for fatigue life estimation of components subjected to Gaussian random loads are no longer applicable in case the random load is non-Gaussian. Utilizing a corrective coefficient for the Gaussian damage based on some non-normality indexes [18], [19], [20], [21], [22] is the most widely used solution in the literature. The fatigue damage $D_{\mathrm{ng}}$ resulting from the non-Gaussian stress time history can then be calculated by multiplying the Gaussian damage $D_{g}$, which is known if the stress PSD is known, by the corrective non-Gaussianity coefficient $\lambda_{\mathrm{ng}}$.

(6) $$\begin{array}{ccc} D_{\mathrm{ng}}\; & =\; & \lambda_{\mathrm{ng}}\;D_{g} \end{array}$$

Kihl et al. [18] observed that in specific conditions, $\lambda_{\mathrm{ng}}$ may take on very high values, exceeding 10. They demonstrated that the main influence on the damage evaluation inaccuracy is connected to the process's kurtosis index $k_{u}$ and the material's Wöhler curve slope *m*. They also carried out experiments on welded specimens to verify the exponential growth of the $\lambda_{\mathrm{ng}}$ coefficient with kurtosis and Wöhler curve slope. Other authors managed to provide explicit formulations for the corrective non-Gaussianity coefficient, mainly based on the main non-Gaussianity indexes (skewness $s_{k}$ and kurtosis $k_{u}$) and on the slope of the material's Wöhler curve. Winterstein [19] and Wang [20] proposed two simple polynomial relationships, shown in equations (7) and (8) respectively, both obtained by exhaustive simulations of non-Gaussian time histories produced through the use of an appropriate non-linear transformation

(7) $$\begin{array}{ccc} \lambda_{\mathrm{ng}}\; & =\; & {\left(\frac{\sqrt{\pi }\;k}{2\Gamma (1+|\nu |)}\right)}^{m}(\frac{\Gamma (1+m|\nu |)}{\Gamma (1+m/2)})\\ k\; & =\; & \frac{1}{\sqrt{1+2h_{3}^{2}+6h_{4}^{2}}}\\ \nu \; & =\; & \sqrt{\frac{4}{\pi }(1+h_{2}+h_{4})-1}\\ h_{1}\; & =\; & \frac{s_{k}}{6}\\ h_{2}\; & =\; & \frac{k_{u}-3}{24}\\ h_{3}\; & =\; & \frac{s_{k}}{6(1+6h_{4})}\\ h_{4}\; & =\; & \frac{\sqrt{1+1.5(k_{u}-3)}-1}{18} \end{array}$$

(8) $$\begin{array}{ccc} \lambda_{\mathrm{ng}}\; & =\; & k^{m}(1+m(m-1)h_{2}+m(m+1)kh_{1}\frac{\sigma }{S_{ut}}) \end{array}$$

Basing on both numerical and experimental data, Braccesi et al. [21] and Cianetti et al. [22] proposed two improved formulations of the non-Gaussianity corrective coefficient $\lambda_{\mathrm{ng}}$, the first of which (equation (9)) is valid for loads with small kurtosis values and non-zero skewness, while the second (equation (10)) is suitable for non-Gaussian signals with high kurtosis and zero skewness.

(9) $$\begin{array}{ccc} \lambda_{\mathrm{ng}}\; & =\; & \exp(\frac{m^{3/2}}{\pi }(\frac{k_{u}-3}{5}-\frac{s_{k}^{2}}{4})) \end{array}$$

(10) $$\begin{array}{ccc} \lambda_{\mathrm{ng}}\; & =\; & \exp(\frac{m^{3/2}}{\pi (0.156+0.416k_{u})}(\frac{k_{u}-3}{5})) \end{array}$$

It is worth noting that the corrective coefficients listed above utilize the parameters of skewness and kurtosis regardless of the method used to generate these non-Gaussian values, yielding satisfactory results. Therefore, while it has been noted that skewness and kurtosis parameters alone are insufficient to unambiguously describe a non-Gaussian random signal (i.e., non-Gaussian signals with the same skewness and kurtosis values but different probability distributions can be obtained [36], [37]), it can still be asserted that these values represent the two most relevant coefficients to be considered for the calculation of damage induced by a non-Gaussian random load. This assertion holds true irrespective of the specific origin of the non-Gaussianity of such a load [35].

According to the formulations above, the calculation of the corrective coefficients requires the knowledge of the skewness and kurtosis values of the stress response of the system. This, in turn, would require an analysis of the stress history in the time domain, thus significantly reducing the computational advantages obtainable from an analysis performed entirely in the frequency domain, only knowing the input signal properties and the system parameters [25].

## 3 Numerical simulations

From what has been said in the previous paragraphs, it is clear that there is potential for further study of the response of a mechanical system at inputs characterized by varying degrees of non-Gaussianity, so that the skewness and kurtosis values of the stress on a component can be predicted directly from the probability distribution properties of the input load and from the system parameters. To this end, an extensive campaign of numerical simulations aimed at evaluating the influence of several parameters on the degree of non-Gaussianity of the response was conducted in this work. The dynamical system under consideration is a simple single-dof mass-spring-damper system, as it is the foundation of classical mechanics and allows simple results to be obtained, yet they can be easily extended to more complex systems.

The first step for numerical simulation is to generate a non-Gaussian random input signal from a desired PSD. This involves creating a Gaussian random signal and then transforming it into a non-Gaussian signal through a nonlinear transformation. The coefficients of the Fourier transform $c_{i}$ can be used to generate a Gaussian signal by applying equation (11).

(11) $$\begin{array}{ccc} x(t)\; & =\; & \frac{1}{N}\sum_{i=0}^{N-1}c_{i}\;e^{\frac{j2\pi i}{N}} \end{array}$$

The coefficients $c_{i}$ can then be calculated using equations (12) to ensure that the load has the desired PSD. In order to generate a signal with Gaussian characteristics, it is important to carefully select the phases $\psi_{i}$ as independent and uniformly distributed within the interval [0,2π].

(12) $$\begin{array}{ccc} |c_{i}{|}^{2}\; & =\; & \frac{1}{2}PSD(i\;\Delta f)\;\Delta f\\ c_{i}\; & =\; & |c_{i}|\;e^{j\;\psi_{i}} \end{array}$$

After generating the Gaussian random signal, it needs to be transformed into a non-Gaussian load using a non-linear conversion applied to the signal. This is based on the assumption that a Gaussian signal x(t) is correlated with a non-Gaussian process y(t) according to equation (13).

(13) $$\begin{array}{ccc} y(t)\; & =\; & G(x(t)) \end{array}$$

It should be noted that there are various non-linear transformations that can be used to create a non-Gaussian signal. One option is to apply a threshold function, where any values below a certain threshold are set to zero; alternatively, different non-linear functions can be used, such as the sigmoid function. However, for the purpose of this study, the chosen method for generating non-Gaussian signals was the corrected formula proposed by Winterstein in 1994 [38]. The reason under this choice lies in its prevalence in the literature as the most widely adopted method for characterizing non-Gaussian random signals, demonstrating robust fitting capabilities to real loads of such nature. Furthermore, this approach allows for the generation of non-Gaussian signals while maintaining the stationarity of the original signal — an attribute not consistently retained by alternative methods, such as those involving the modulation of signal amplitude through a modifying function [39]. The Winterstein formula is modeled as a cubic Hermite polynomial function and can estimate the transformation function based on the first four central moments of the non-Gaussian signal distribution (*μ*, *σ*, $s_{k}$ and $k_{u}$). If the signal is leptokurtic, with a kurtosis higher than 3, the Winterstein formulation (equation (14)) provides a direct transformation from a Gaussian to a non-Gaussian load.

(14) $$\begin{array}{ccc} G(x)\; & =\; & \mu \;\frac{\sigma (x+c_{1}(x^{2}-1)+c_{2}(x^{3}-3x))}{\sqrt{1+2c_{1}^{2}+6c_{2}^{2}}}\\ c_{1}\; & =\; & \frac{s_{k}}{6}(\frac{1-0.015|s_{k}|+0.3s_{k}^{2}}{1+0.2(k_{u}-3)})\\ c_{2}\; & =\; & 0.1({\left(1+1.25(k_{u}-3)\right)}^{1/3}-1){\left(1-\frac{1.43s_{k}^{2}}{ku-3}\right)}^{1-0.1k_{u}^{0.8}} \end{array}$$

Once the non-Gaussian signal has been generated, numerical simulation of the single-dof system can be performed, and the response analyzed to determine the values of skewness and kurtosis. These two values are of interest and are monitored in the numerical campaign. In particular, the variation of the kurtosis and skewness values was evaluated by considering the effect of several input parameters, which include both the input load (its frequency content and the characteristics of its probability distribution) and the characteristics of the excited system (eigenfrequency and damping).

**Figure 1:**
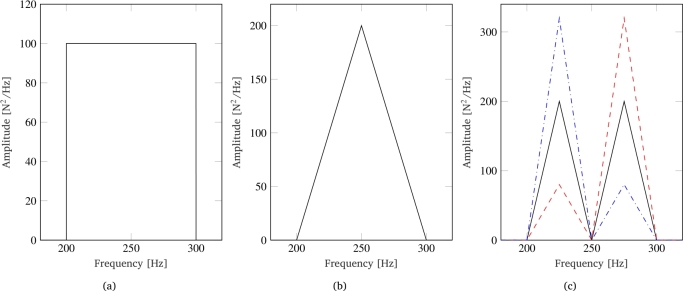
PSD used for numerical simulations: rectangular (a), triangular (b) and bimodal (c) PSD.

Most simulations were carried out using rectangular-shaped PSDs, which distribute the signal power uniformly over a frequency range of variable width, but always centered around the eigenfrequency of the dynamic system. Additionally, a number of simulations were performed considering different PSD shapes to evaluate their influence on the system output and to approximate a wider range of realistic load PSD shapes. The tested PSDs, shown in Fig. 1 along with the rectangular one, include a triangular PSD and several bimodal PSDs with signal power distributed at different ratios on frequencies above or below the system's eigenfrequency. It is worth to note that while the PSD shapes presented may not precisely replicate those found in real-world measurements, they serve as simplifications for the specific research purposes of this study. Such simplifications are commonly employed in the literature to facilitate analysis and interpretation of results in frequency fatigue. Regarding the characteristics of the input load probability distribution, we have seen how the Winterstein equation (eq. (14)) allows us to control all four central moments (mean, standard deviation, skewness, and kurtosis). However, the values of the mean and standard deviation were kept constant for all simulations, set to 0 N and 100 N respectively. This is because the mean and standard deviation of the signal represent a simple translation and scaling of the signal, which is uniform over its entire duration. Therefore, despite their high impact on the estimation of fatigue life, their variation has no influence on the two parameters of interest for this study. In contrast, the input values of skewness and kurtosis have a significant influence on the degree of non-Gaussianity of the system response. Given that experimental acquisitions coming from mechanical design purposes often yield kurtosis values greater than 3 (e.g. softening non-Gaussian loads), in this study we specifically focused on investigating softening non-Gaussian cases. For this reason, kurtosis values between 4 and 10 were considered, with increments of 1, and skewness values between 0 and 1 were considered, with increments of 0.2. Some simulations were also performed with negative skewness values to verify the robustness of our findings across a range of skewness values.

Concerning the characteristics of the dynamic system, the parameters investigated for their influence were the natural frequency and damping of the system. The eigenfrequency of the system was varied in some cases, but always in relation to the frequency content of the input signal's PSD. Anyway, it is important to clarify that there is no influence of the input signal on the eigenfrequency of the dynamic system, which remains solely tied to the mass and stiffness characteristics of the system itself. The goal is to identify a frequency-related parameter that could link the system's eigenfrequency to the frequencies included in the band of the forcing load, without implying a direct dependency relationship between them, and which could represent a characteristic parameter of the problem. On the other hand, damping turned out to be one of the parameters that most strongly influences the behavior of the system's dynamic response in terms of non-Gaussianity. For this reason, numerous simulations were performed with various damping values, ranging from a very low damping coefficient of 0.1%, up to critical damping.

The final achievement of this numerical campaign, therefore, is intended to be a mapping of the skewness and kurtosis values of the system's response as a function of the many load and system input parameters that have just been described.

## 4 Results and discussion

### 4.1 Damping influence

**Figure 2:**
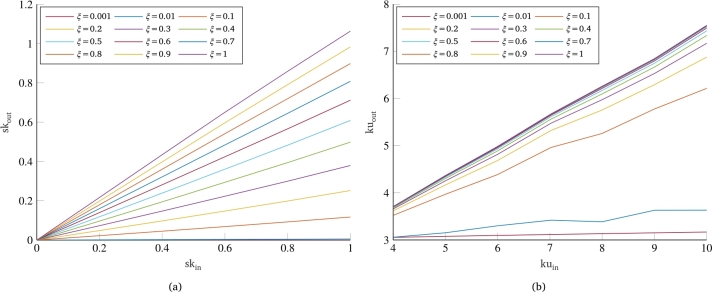
Influence of input non-normality indexes on non-Gaussianity of the output signal: skewness (a) and kurtosis (b).

Fig. 2 shows some results of the numerical simulations performed in this work. In particular, the skewness and kurtosis values of the dynamic response as a function of the same parameters referring to the input signal are shown for various values of the damping coefficient *ξ* of the single-dof system. All curves show that there is a linear relationship between the degree of non-Gaussianity of the system output and the degree of non-Gaussianity of the input load. In addition, an important dependence on damping is observed for both parameters: For very low values of damping, consistent with typical damping ratios of metallic structures in real vibration testing, the response of the system turns out to be Gaussian regardless of the degree of non-Gaussianity of the load to which it is subjected (curves with ξ=0.001 and ξ=0.01), thus confirming the results already found in the literature; conversely, an increase in the damping coefficient of the system results in a greater degree of non-Gaussianity of the system's response. This result can be explained by considering that the non-Gaussianity of a signal is related to its frequency content in terms of the phase of the Fourier transform [36]. Indeed, the PSD does not provide any information about the Gaussian or non-Gaussian nature of a signal, as it only corresponds to the squared magnitude of the transform, losing information about the phase of the different frequencies. Therefore, for dynamic systems with very low damping, the input frequency is heavily filtered, resulting in a very narrow band response around the system's eigenfrequency. Consequently, the non-Gaussianity of the input signal, which was distributed across various frequencies in the PSD bandwidth, is almost completely eliminated, along with the frequencies far from the system's eigenfrequency.

**Figure 3:**
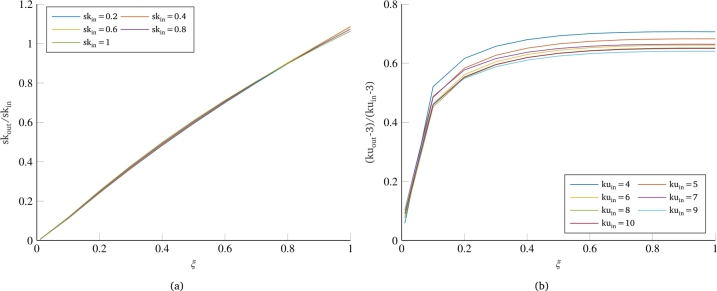
Effect of damping coefficient on the normalized values of skewness (a) and kurtosis (b).

However, differences are also observed between skewness behavior (Fig. 2a) and kurtosis behavior (Fig. 2b). At first, the kurtosis of the response always undergoes a reduction from the input kurtosis for all values of the damping coefficient. In contrast, the skewness exhibits curves with almost unitary slope for very high damping (90-100%), which means that the skewness value in output does not undergo variation from the input value. A further difference is found in the dependence of these two parameters on the damping of the system. The curves for various dampings in the case of skewness appear to be equispaced, suggesting an approximately linear dependence of this parameter on the damping of the system. Conversely, the kurtosis curves relating various dampings show an initially very wide spacing, which then decreases for very high damping coefficients, more similar to an asymptotic trend. These considerations are confirmed by Fig. 3, which shows the trends of skewness and kurtosis values normalized with respect to the same values in input as a function of the damping coefficient of the single-dof system. For skewness, this equals a simple ratio of the skewness of the response of the dynamic system to the skewness of the input load; for kurtosis, on the other hand, it is necessary to compute the ratio between the excesses of kurtosis with respect to the Gaussian case, i.e., reducing by 3 the kurtosis value of both the input and the output of the system. Concerning the dependence on damping, Fig. 3a confirms the prediction that skewness has an approximately linear dependence on the damping coefficient of the system. Similarly, Fig. 3b also confirms what was said for kurtosis, for which the curves have a strongly nonlinear trend, with a very rapid increase in the initial part and a sort of plateau for higher dampings.

**Figure 4:**
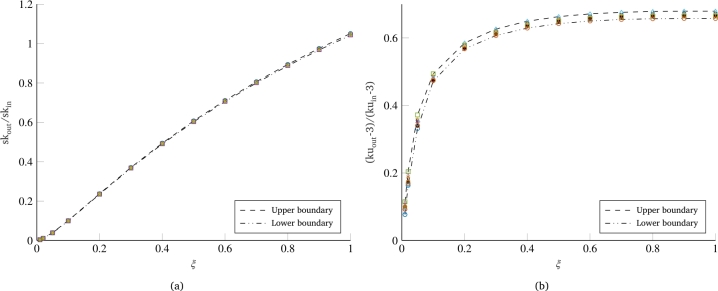
Example of the scatter and uncertainty associated with the normalized non-normality indices curves: skewness (a) and kurtosis (b).

One further consideration can be made regarding the overlapping of the curves shown in Fig. 3. All the curves in Fig. 3a are almost perfectly overlapping, signifying that the percentage reduction of the skewness value as a function of the damping coefficient of the system remains the same regardless of the starting skewness value of the load that stresses the system. As for kurtosis, in Fig. 3b, the overlapping of the curves is not as pronounced as in the case of skewness. Looking at the positioning of the curves, a greater reduction of the output kurtosis is observed for cases with a higher value of the kurtosis of the random input load. In any case, the maximum difference between the reported curves remains below 10%, also showing the same trend as a function of system damping. In this regard, it is important to note that we are working with random signals, and consequently, each of the curves represented here can be associated with a certain dispersion or uncertainty. To get an idea of the confidence level of the curves, a significant number of simulations were performed with the same problem parameters but starting from different random signals (having the same PSD). Fig. 4, where the dashed curves represent the extremes within which all simulation results are included, shows how the uncertainty on the skewness results is practically negligible (Fig. 4a), while there is some dispersion on the curves related to the kurtosis, which still remains below 4% (Fig. 4b).

It is worth noting once again that the results of our study primarily pertain to the investigation of softening non-Gaussian cases. However, it is pertinent to acknowledge that certain conclusions drawn, particularly regarding the influence of damping on system response, may hold relevance for hardening non-Gaussian loads as well. Specifically, our findings suggest that for dynamic systems characterized by very low damping values, the response may tend towards Gaussian behavior irrespective of the load's kurtosis, even in cases where the kurtosis is less than 3. This phenomenon arises due to the inherent relationship between non-Gaussianity and the phase of the load's Fourier transform, coupled with the filtering effect exerted by dynamic systems with minimal damping.

### 4.2 Cross influence between kurtosis and skewness

**Figure 5:**
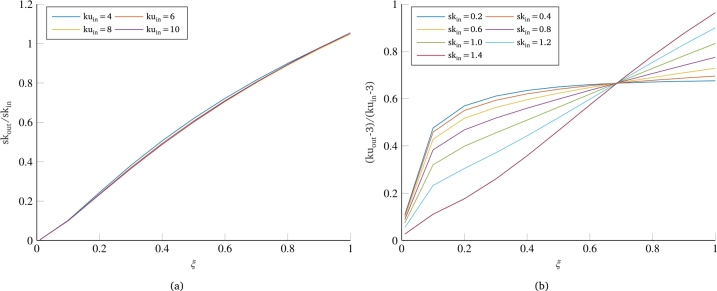
Effect of different input kurtosis values on the skewness reduction (a) and of different values of input skewness on kurtosis reduction (b) between input and output signals.

Fig. 5 shows the cross-effect that the two non-Gaussianity parameters have on each other. In other words, several curves are presented showing how different values of kurtosis of the input load affect the reduction of skewness as a function of damping (Fig. 5a) and vice-versa (Fig. 5b). Again, the curves related to skewness are almost perfectly overlapping, demonstrating that the kurtosis value of the load has no influence on the other non-Gaussianity parameter. Conversely, Fig. 5b shows how the input skewness greatly influences how the kurtosis parameter is reduced according to the damping of the system. In particular, there is a considerable variation in the shape of the curves, which reduce their curvature as the skewness value increases until they reach an almost linear shape for higher skewness values. In addition, an interesting behavior lies in the fact that all curves intersect at the same point, for a damping coefficient of about 70%, which thus seems to represent a node for the displayed curves.

**Figure 6:**
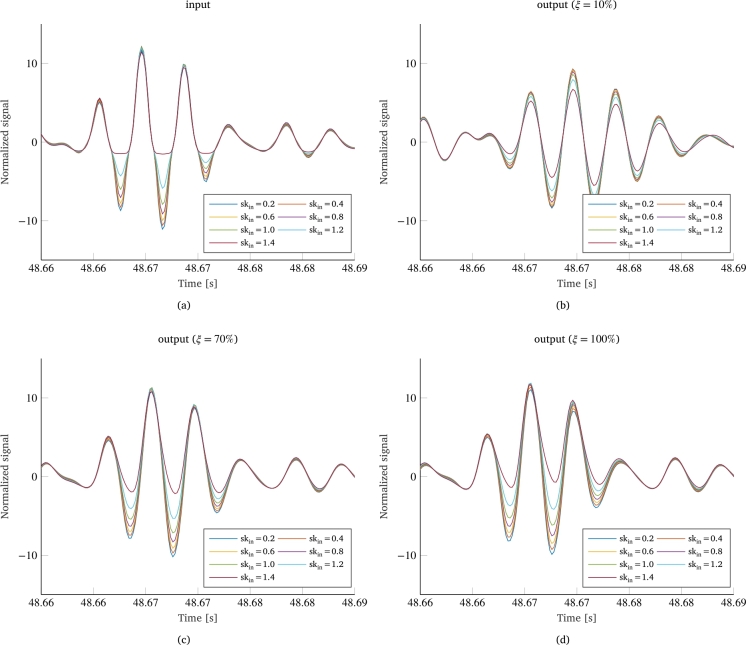
Configurations of the same segment of a signal for various skewness values in four different scenarios: input signal (a), output signal for a damping coefficient of 10% (b), 70% (c), and 100% (d).

To better understand this behavior, Fig. 6 shows different configurations of the same segment of a signal for various skewness values in four different scenarios: the input signal (Fig. 6a), and the response for a damping coefficient of 10% (Fig. 6b), 70% (Fig. 6c), and 100% (Fig. 6d). Observing the input signal, and considering that the signals started with the same kurtosis value, it can be noted that signals with different skewness values but the same kurtosis value exhibit almost overlapping positive peaks. For relatively low damping, it has been observed that the system's mechanical response tends to reduce both the kurtosis and skewness of the output signal, bringing it closer to a Gaussian distribution. A reduction in skewness tends to make the signal more symmetrical, shifting both the positive and negative peaks in the opposite direction of the asymmetry of the input signal. On the other hand, a reduction in kurtosis tends to reduce the length of the tails of the signal distribution, thereby reducing the magnitude of the peaks that deviate the most from the signal mean, regardless of whether they are positive or negative. However, for low damping the reduction in skewness is greater than the reduction in kurtosis, resulting in an overall downward shift of the peaks, which tend to distribute with greater symmetry around the signal mean. In this situation, the positive peaks of signals with higher skewness values end up being lower than the others, resulting in a lower kurtosis value (Fig. 6b). For damping values close to the critical damping, the opposite happens: the skewness of the various signals does not vary, while the kurtosis still undergoes a slight reduction. Therefore, in the absence of a shift in the signal towards greater symmetry, the reduction in kurtosis results in a greater reduction of the positive peaks for signals with lower skewness values, while in cases of very high skewness, the positive peaks remain almost unchanged, resulting in a higher kurtosis value (Fig. 6d). In the midst of these opposing cases, there will certainly be an intermediate case where the positive peaks of the various signals maintain the same value. In Fig. 6c, it is observed that the behavior of the various dynamic responses is actually similar to that of the input signals, resulting in the same kurtosis value for all signals (although both the kurtosis and skewness values have undergone a reduction between the input and output of the system).

### 4.3 Frequency-based invariant

As previously said, one of the goals of this work is to identify a frequency-related parameter that represents an invariant of the problem. That is to say that so far as the frequencies involved in the simulation change (i.e. the signal's bandwidth or the system's eigenfrequency), but this parameter, which links the system's eigenfrequency to the PSD bandwidth, doesn't vary, the results in terms of non-Gaussianity reduction of the system response remain the same. The desired parameter, which was named *Normalized Bandwidth Factor* $r_{f}$, was found to be the ratio between the bandwidth $\Delta f=f_{max}-f_{min}$ of the PSD and the value of the system's eigenfrequency $f_{n}$ (equation (15)).

(15) $$\begin{array}{ccc} r_{f}\; & =\; & \frac{f_{max}-f_{min}}{f_{n}} \end{array}$$

**Figure 7:**
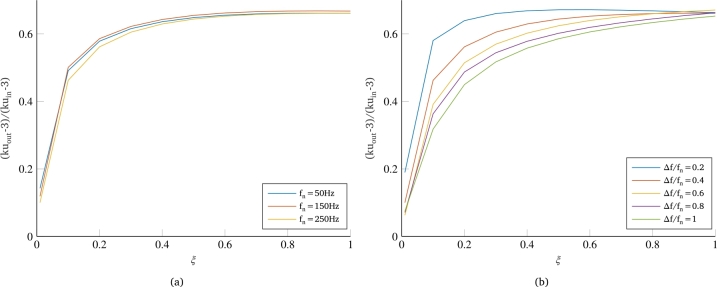
Effect of variations in PSD frequency bandwidth and eigenfrequency of the system on kurtosis reduction: behavior of the Normalized Bandwidth Factor as the frequency-based invariant parameter for non-Gaussianity reduction (a) and effect of a variation of Normalized Bandwidth Factor on the kurtosis reduction (b).

Fig. 7a shows the results from simulations performed for systems with three different eigenfrequencies, but each simulation was carried out with a PSD that grants to always have the same ratio $r_{f}$. The almost perfect overlap between the three curves related to kurtosis reduction confirms the goodness of the choice of ratio $r_{f}$ as the frequency-based invariant parameter for non-Gaussianity reduction.

Further confirmation is obtained from Fig. 7b, which shows the results of simulations performed with PSDs of variable amplitude, but with systems having the same eigenfrequency, thus having different values of the normalized bandwidth factor. The curves obtained for different values of $r_{f}$ show significantly different trends, but with a recognizable pattern that allows to determine the effect that the ratio of PSD bandwidth to system eigenfrequency has on the non-Gaussianity of the dynamic response. In particular, although the final value (i.e., for damping of 100%) remains the same for all curves, an increase in the value of $r_{f}$ results in a decrease in the slope of curves at low damping coefficients and a more gradual increase in the output kurtosis as the damping of the single-dof system increases. This behavior is consistent with the considerations made above regarding the correlation between the non-Gaussianity of a signal and the phase of the Fourier transform of that signal. Indeed, for higher values of the normalized bandwidth factor, we can say that the non-Gaussianity of the input is distributed over a wider range of frequencies, and consequently, the percentage of those frequencies that are attenuated in favor of those closer to the system's eigenfrequency is also greater for intermediate values of the system's damping, causing a greater reduction in the non-Gaussianity of the dynamic response.

### 4.4 Influence of the PSD

**Figure 8:**
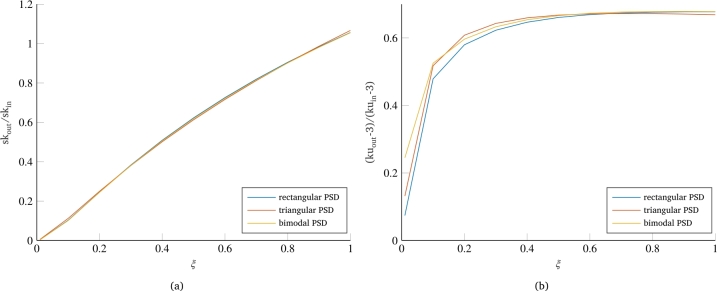
Effect of PSD shape on non-Gaussianity reduction: skewness (a) and kurtosis(b).

Regarding the influence of the PSD shape on the two non-Gaussianity parameters, Fig. 8 shows the results for the three different tested PSD shapes, where the bimodal PSD is characterized by an equal distribution of signal power on the two frequencies (black PSD in Fig. 1c). While the skewness shows no significant variation with respect to the PSD shape (Fig. 8b), the kurtosis curves (Fig. 8b) show a small but noticeable variation. In particular, the curve for the triangular PSD shows a more abrupt increase in the kurtosis value at low damping coefficient values. This result is consistent with the previous findings since the triangular PSD has a greater relevance to the central frequencies close to the system's eigenfrequency. Consequently, even for low damping values, the output signal maintains most of the relevant frequencies of the input load, on whose phases the non-Gaussianity of the signal is distributed. In contrast, the bimodal PSD has low PSD values for frequencies closer to the system's eigenfrequency. As a result, it is observed that the corresponding curve, although starting from a higher value, shows a lower slope compared to the curves of both other PSDs.

**Figure 9:**
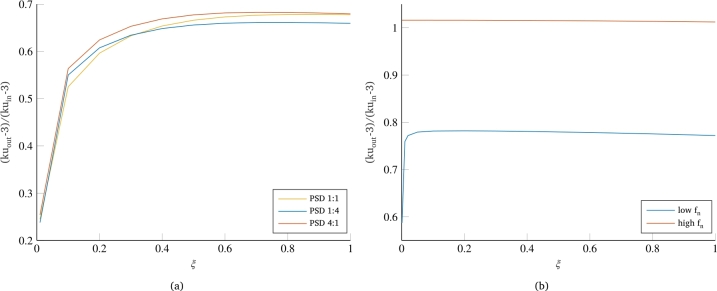
Effect of power distribution in bimodal PSDs on the kurtosis reduction (a) and extreme cases of the positioning of the system's eigenfrequency with respect to the PSD frequency span (b).

One last consideration can be done regarding how different distribution of signal power over the frequencies of the bimodal PSD affects the kurtosis value of the system output. Fig. 9a shows the results for the bimodal PSD with equidistributed signal power (PSD 1:1), with power distributed in favor of frequencies higher than the eigenfrequency of the system (PSD 1:4) and with power distributed in favor of frequencies lower than the $f_{n}$ of the system. Although relatively similar, the curves show a pattern that is connectable to the curves of Fig. 9b, concerning two extreme cases with the eigenfrequency of the system respectively much lower or much higher (i.e. at least an order of magnitude) than the frequencies characterizing the input load. In fact, both of the two curves with uneven distribution of signal power over the bimodal frequencies have a higher slope in the initial part of the curve, which results in a higher value of output kurtosis even for very low damping. On the other hand, in the asymptotic part of the curve for high damping, the two curves differ by showing a different final value. This value is higher in the case of PSD with power shifted to the frequencies lower than the system's eigenfrequency, while it is lower in the opposite case. Similarly, the two curves of Fig. 9b show that in the case of eigenfrequency much higher than the frequencies of the signal PSD, the system actually behaves as a rigid system and the output value of kurtosis is almost identical to that of the input load. Conversely, the case with eigenfrequency much lower than the frequencies of the PSD of the load, although presenting a higher output value of kurtosis than the curves in Fig. 9a, still maintains some reductions in the output value of kurtosis.

## 5 Conclusions

The paper presents a numerical study on the non-Gaussianity of the response of a single-dof mass-spring-damper system subjected to non-Gaussian random loads. The main focus of the study is to evaluate the influence of various input and system parameters on the skewness and kurtosis of the response. The main findings of the study are summarized and listed below:

• There exists an almost linear relationship between the degree of non-Gaussianity of the input load and the system output. This finding is probably closely tied to the fact that the analyzed model is a linear system. Indeed, this probably does not apply in the context of nonlinear systems, since it is conceivable that non-Gaussian outputs could arise even in response to Gaussian-type loads, due to the inherent nonlinearity of the system.
• The damping coefficient of the system has a significant impact on both the skewness and kurtosis of the response. For very low damping values, the response of the system is found to be Gaussian, while an increase in damping leads to a greater degree of non-Gaussianity of the response.
• The cross-effect that skewness and kurtosis have on each other is also investigated. The kurtosis of the input load does not influence the skewness of the system's response, but input skewness significantly affects how the kurtosis parameter decreases with damping.
• A significant number of simulations with identical problem parameters but different random signals was performed in order to evaluate the robustness of the results, confirming negligible uncertainty in skewness results and a dispersion in kurtosis curves below 4%.
• A new parameter is introduced, called the Normalized Bandwidth Factor, that links the system's eigenfrequency to the PSD bandwidth and acts as an invariant of the problem. The study shows that an increase in the Normalized Bandwidth Factor results in a decrease in the slope of curves at low damping coefficients and a more gradual increase in the output kurtosis as the damping of the system increases.
• Finally, the study also evaluates the qualitative effect of different PSD shapes on the non-normality indexes. In particular, the tested PSDs include a triangular PSD and several bimodal PSDs with signal power distributed at different ratios on frequencies above or below the system's eigenfrequency.

It is noteworthy that the results presented in this study were obtained using stationary non-Gaussian random signals generated through the Winterstein transformation [38]. Therefore, these findings are, at least quantitatively, specific to loads of this nature and may not be entirely generalizable to every type of non-Gaussian signal. Nevertheless, it is crucial to emphasize that the chosen method for load generation was selected in this study because it is the most commonly employed technique in the literature for describing non-Gaussian random loads derived from real-world scenarios. Furthermore, in the case of stationary non-Gaussian random loads, the non-Gaussianity of the signal is linked to the phase of the Fourier transform (rather than its magnitude, as the Gaussianity of a signal is not discernible from its power spectral density) [36]. Therefore, it is reasonable to anticipate analogous results, at least qualitatively (e.g., the influence of system damping and the effect of the Normalized Bandwidth Factor), even for other methods of generating non-Gaussian signals.

Furthermore, while a comprehensive method for systematically and accurately conducting frequency domain analysis under all conditions is still lacking, this study has identified specific scenarios where such an approach shows promise. Notably, in systems characterized by very low damping (e.g., ξ≃0.01 or lower), the stress response tends towards Gaussian behavior, enabling accurate fatigue estimation using established frequency domain methods such as Bendat's and Dirlik's. Furthermore, even in systems with significantly high damping, where stress displays non-Gaussian characteristics, frequency domain fatigue verification remains feasible. By employing corrective formulas outlined in Section 2, with a kurtosis value cautiously chosen to be approximately 0.7-0.8 times that of the load, reasonably accurate fatigue assessments can be achieved. Lastly, while this approach may introduce a slightly larger error in damage calculation, it can also be applied to systems with moderate damping, where stress kurtosis varies widely between cases. Despite the ongoing need for further research, these findings underscore the potential advancement of frequency domain fatigue analysis methods for non-Gaussian loadings.

Overall, this study contributes to a better understanding of the non-Gaussianity of the response of dynamic systems subjected to stationary non-Gaussian random loads. The findings of this study are expected to have significant implications for structural engineering applications where non-Gaussian random loads play a critical role in the system's response, allowing fatigue analyses to be conducted entirely in the frequency domain with significant time-saving. Future work will extend the analysis to the dynamic response of systems subjected to non-stationary random loads, broadening the understanding of non-Gaussianity and non-stationarity in dynamic systems.

## CRediT authorship contribution statement

**Michele Sgamma:** Writing – review & editing, Writing – original draft, Software, Methodology, Formal analysis, Conceptualization. **Massimiliano Palmieri:** Writing – review & editing, Software, Methodology. **Michele Barsanti:** Writing – review & editing. **Francesco Bucchi:** Writing – review & editing, Supervision. **Filippo Cianetti:** Writing – review & editing, Supervision, Conceptualization. **Francesco Frendo:** Writing – review & editing, Supervision.

## Declaration of Competing Interest

The authors declare the following financial interests/personal relationships which may be considered as potential competing interests: Francesco Frendo reports financial support was provided by Government of Italy Ministry of Education University and Research.

## Data Availability

Data will be made available on request.

## References

[1] Rychlik I. (1987). A new definition of the rainflow cycle counting method. Int. J. Fatigue.

[2] Bendat J.S. (1964).

[3] Wirsching P.H., Light M.C. (1980). Fatigue under wide band random stresses. J. Struct. Div..

[4] Ortiz K., Chen N. (1987). Proc. Fifth Int. Conf. on Applications of Statistics and Probability in Soil and Struct.

[5] Larsen C.E., Lutes L.D. (1991). Stochastic Structural Dynamics 2: New Practical Applications Second International Conference on Stochastic Structural Dynamics.

[6] Benasciutti D., Tovo R. (2005). Spectral methods for lifetime prediction under wide-band stationary random processes. Int. J. Fatigue.

[7] Dirlik T. (1985).

[8] Zhao W., Baker M.J. (1992). On the probability density function of rainflow stress range for stationary Gaussian processes. Int. J. Fatigue.

[9] Sakai S., Okamura H. (1995). On the distribution of rainflow range for Gaussian random processes with bimodal psd. JSME Int. J. Ser. a Mech. Mater. Eng..

[10] Benasciutti D., Tovo R. (2005).

[11] Sgamma M., Bucchi F., Frendo F. (2022).

[12] Sgamma M., Chiocca A., Bucchi F., Frendo F. (2022). Frequency analysis of random fatigue: setup for an experimental study. Appl. Res..

[13] Niesłony A., Rŭžička M., Papuga J., Hodr A., Balda M., Svoboda J. (2012). Fatigue life prediction for broad-band multiaxial loading with various psd curve shapes. Int. J. Fatigue.

[14] Xu F., Chen Z., Ahlin K. (2024). Research on vehicle vibration fatigue damage potential under non-Gaussian road profile excitation. Shock Vib..

[15] Rouillard V., Sek M.A. (2001). Simulation of non-stationary vehicle vibrations, proceedings of the institution of mechanical engineers, part D. J. Automob. Eng..

[16] Amiri K., Mulu B., Raisee M., Cervantes M. (2014).

[17] Gao Z., Moan T. (2007). Fatigue damage induced by nongaussian bimodal wave loading in mooring lines. Appl. Ocean Res..

[18] Kihl D., Sarkani S., Beach J. (1995). Stochastic fatigue damage accumulation under broadband loadings. Int. J. Fatigue.

[19] Winterstein S.R. (1988). Nonlinear vibration models for extremes and fatigue. J. Eng. Mech..

[20] Wang X., Sun J. (2005). Effect of skewness on fatigue life with mean stress correction. J. Sound Vib..

[21] Braccesi C., Cianetti F., Lori G., Pioli D. (2009). The frequency domain approach in virtual fatigue estimation of non-linear systems: the problem of non-Gaussian states of stress. Int. J. Fatigue.

[22] Cianetti F., Palmieri M., Braccesi C., Morettini G. (2018). Correction formula approach to evaluate fatigue damage induced by non-Gaussian stress state. Proc. Struct. Integrity.

[23] Gao S., Zheng X.Y., Wang B., Zhao S., Li W. (2021). Assessment of fatigue damage induced by non-Gaussian bimodal processes with emphasis on spectral methods. Ocean Eng..

[24] Yuan K., Sun Z. (2023). A spectral method for accurate evaluation of fatigue damage induced by wide-band non-Gaussian random processes. Ocean Eng..

[25] Cianetti F., Palmieri M., Slavič J., Braccesi C., Morettini G. (2017). The effort of the dynamic simulation on the fatigue damage evaluation of flexible mechanical systems loaded by non-Gaussian and non stationary loads. Int. J. Fatigue.

[26] Rizzi S.A., Przekop A., Turner T.L. (2011). EURODYN2011-8th International Conference on Structural Dynamics.

[27] F. Kihm, S. Rizzi, N. Ferguson, A. Halfpenny, Understanding how kurtosis is transferred from input acceleration to stress response and it's influence on fatigue life, 2013.

[28] Palmieri M., Česnik M., Slavič J., Cianetti F., Boltežar M. (2017). Non-Gaussianity and non-stationarity in vibration fatigue. Int. J. Fatigue.

[29] Braccesi C., Cianetti F., Palmieri M., Zucca G. (2018). The importance of dynamic behaviour of vibrating systems on the response in case of non-Gaussian random excitations. Proc. Struct. Integrity.

[30] Trapp A., Hollweck F., Wolfsteiner P. (2022). On the transmission of non-Gaussian random loading through linear structures. Proc. Struct. Integrity.

[31] Fan W., Sheng X., Li Z., Sun Y. (2022). The higher-order analysis method of statistics analysis for response of linear structure under stationary non-Gaussian excitation. Mech. Syst. Signal Process..

[32] Rice S.O. (1944). Mathematical analysis of random noise. Bell Syst. Tech. J..

[33] Braccesi C., Cianetti F., Lori G., Pioli D. (2005). Fatigue behaviour analysis of mechanical components subject to random bimodal stress process: frequency domain approach. Int. J. Fatigue.

[34] Benasciutti D., Tovo R. (2006). Comparison of spectral methods for fatigue analysis of broad-band Gaussian random processes. Probab. Eng. Mech..

[35] Wang M., Wang C., Guan Y. (2017). A comparative study on damage analysis between Gaussian and non-Gaussian random vibration. Vibroeng. Proc..

[36] Zheng R., Guoping C., Huaihai C. (2021). Stationary non-Gaussian random vibration control: a review. Chin. J. Aeronaut..

[37] Niesłony A., Böhm M., Owsiński R. (2021). Crest factor and kurtosis parameter under vibrational random loading. Int. J. Fatigue.

[38] Winterstein S.R., Ude T.C., Kleiven G. (1994). Springing and slow-drift responses: predicted extremes and fatigue vs. simulation. Proc. BOSS-94.

[39] Trapp A., Makua M.J., Wolfsteiner P. (2019). Fatigue assessment of amplitude-modulated non-stationary random vibration loading. Proc. Struct. Integrity.

